# Do extra compulsory physical education lessons mean more physically active children - findings from the childhood health, activity, and motor performance school study Denmark (The CHAMPS-study DK)

**DOI:** 10.1186/s12966-014-0121-0

**Published:** 2014-09-24

**Authors:** Niels Christian Møller, Jakob Tarp, Eva Friis Kamelarczyk, Jan Christian Brønd, Heidi Klakk, Niels Wedderkopp

**Affiliations:** Centre of Research in Childhood Health, Institute for Sport Science and Clinical Biomechanics, University of Southern Denmark, Odense, Denmark; Spine Centre of Southern Denmark, Hospital Lillebaelt, Middelfart, Denmark

**Keywords:** Physical activity, Physical education, School-based, Organized sports, Children, Objective monitoring, Accelerometry, CHAMPS-study DK

## Abstract

**Background:**

Primarily, this study aims to examine whether children attending sports schools are more active than their counterpart attending normal schools. Secondary, the study aims to examine if physical activity (PA) levels in specific domains differ across school types. Finally, potential modifications by status of overweight/obesity and poor cardio-respiratory fitness are examined.

**Methods:**

Participants were from the first part of the CHAMPS-study DK, which included approximately 1200 children attending the 0th – 6th grade. At the sports schools, the mandatory physical education (PE) program was increased from 2 to 6 weekly lessons over a 3-year period. Children attending normal schools were offered the standard 2 PE lessons. PA was assessed at two different occasions with the GT3X ActiGraph accelerometer, once during winter in 2009/10 and once during summer/fall in 2010. Leisure time organized sports participation was quantified by SMS track. Based on baseline values in 2008, we generated a high-BMI and a low-cardio-respiratory fitness for age and sex group variable.

**Results:**

There were no significant differences in PA levels during total time, PE, or recess between children attending sports schools and normal schools, respectively. However, children, especially boys, attending sports schools were more active during school time than children attending normal schools (girls: β=51, p=0.065; boys: β=113, p<0.001). However, in the leisure time during weekdays children who attended sports schools were less active (girls: β=-41, p=0.004; boys: β=-72, p<0.001) and less involved in leisure time organized sports participation (girls: β=-0.4, p=0.016; boys: β=-0.2, p=0.236) than children who attended normal schools. Examination of modification by baseline status of overweight/obesity and low cardio-respiratory fitness indicated that during PE low fit girls in particular were more active at sports schools.

**Conclusion:**

No differences were revealed in overall PA levels between children attending sports schools and normal schools. Sports schools children were more active than normal schools children during school time, but less active during leisure time. In girls, less organized sports participation at least partly explained the observed differences in PA levels during leisure time across school types. Baseline status of cardio-respiratory fitness modified school type differences in PA levels during PE in girls.

**Electronic supplementary material:**

The online version of this article (doi:10.1186/s12966-014-0121-0) contains supplementary material, which is available to authorized users.

## Background

Physical inactivity has been identified as the fourth leading risk factor for non-communicable diseases accounting for more than 3 million preventable deaths in 2009 [1]. Being physically active is pivotal not only for adults’ health but also holds important health benefits in children and in youth, where low physical activity (PA) and the consequences hereof such as poor physical fitness have been found to relate to other cardiovascular disease risk factors, including overweight/obesity, and mental despair [2]. Low PA, poor physical fitness, and overweight track from childhood through adolescence [3-5] into the adult years [6,7], and recently it has been suggested that PA also plays a role in relation to brain function and learning performance [8,9]. Consequently, PA should ideally be promoted in young age in order to avoid attendant substantial and long-lasting health problems and possibly even improve cognitive function and academic performance.

PA interventions are founded on the convictions that disease prophylaxis is preferable to treatment of disease and that PA is habitual and thus rather stable over time. The school setting as basis for PA interventions seems advantageous since children from all risk groups and all segments of the population go to school and spend most of their day there, and schools have been pointed out to be potentially effective arenas for preventive strategies and promotion of healthy lifestyle [10,11]. However, it is not clear what the most effective school-based strategies are to promote long lasting healthy lifestyle behaviours, and more long-term assessments of the impact of school-based interventions on physical health status measurements combined with PA assessments are important if significant improvements in chronic disease risk are to be realized [10,12]. Extra physical education (PE) as a strategy to increase PA seems appealing since adherence with the program theoretically is high as PE lessons are mandatory for all school children. Therefore, to initiate sports schools, i.e. by increasing the number of PE lessons and intensifying the focus on health and PA, could maybe be a feasible way of optimizing the possibilities of enhancing children’s levels of PA, fitness, and body weight at an early stage of life.

This study relies on data from the Childhood Health Activity and Motor Performance School study – Denmark (CHAMPS-study DK) [13] which examines a wide range of possible effects of extra compulsory PE lessons in a large group of Danish school children. Many of the project evaluation parameters are based on the assumption that children attending schools with extra PE lessons are more physically active compared to children attending normal schools. Optimally, however, final conclusions on the possible health impact of children attending sports schools should be seen in the light of a thorough examination of the primary exposure PA. Previous findings support the notion that often school based PA interventions do not seem to transfer into overall increased PA levels [14]. Therefore, detailed knowledge in terms of how an intervention program in the form of extra PE lessons during school time might cause compensated behavior of the children in other contexts is pivotal for program evaluation and potentially adjustment of future school based PA interventions. This study primarily aims at examining whether children attending sports schools are actually more active than their counterpart attending normal schools. Secondary, the study aims to examine 1) if any potential effect of extra compulsory PE, provided at the sports schools, will be limited to PE lessons or other school domains or can be transferred into an overall positive PA effect, 2) how potential changes in school-time PA will affect leisure time PA levels, 3) if leisure time organized sports participation differs across the two school types, and 4) if PA levels across school types are moderated by baseline status of overweight/obesity or poor cardio-respiratory fitness.

## Methods

### Design and participants

In 2007, the county council in the municipality of Svendborg in Denmark took the decision to provide increased levels of suitable PA in some of their primary schools, with the aim to improve the physical health of the children. All 19 public primary schools in the area were invited to participate in a natural experiment, of which 6 agreed to partake. In Denmark, the primary school consists of one year of mandatory pre-school, referred to as the 0th grade, mandatory elementary school from 1st to 9th grade, and on top of that the 10th grade which is optional. A quasi experimental research program (i.e. The CHAMPS-study DK), with 4 normal schools serving as controls, was subsequently established in order to examine the possible beneficial effects of increased PA. Thereby, the CHAMPS-study DK embraces ten public schools matched according to school size and socio-economical status of the uptake area. Two pairs of sports schools were matched with one normal school since they shared the same uptake area of children. In 2008, 697 sports school children (45% boys and 55% girls attending 0^th^ - 4^th^ grade) and 521 normal school children (50% boys and 50% girls attending 0^th^ - 4^th^ grade) were enrolled in the study. Seven hundred and fifteen sports school children (44% boys and 56% girls attending 2^nd^ - 6^th^ grade) and 514 normal school children (50% boys and 50% girls attending 2^nd^ - 6^th^ grade) participated in the study at the end of the school year in 2011 (Additional file 1). The CHAMPS-study DK has previously been described in details elsewhere [13].

### Intervention

The school leaders and PE teachers were invited to design the set-up for an optimal sports school. Thus a committee consisting of people from these schools worked out a proposition based on an age-related training concept [15]. This concept aims to ensure that all children are trained in a biologically relevant manner depending on their physical and physiological maturity. Briefly, for the age groups included in the present study, the concept primarily focuses on development of fundamental bodily skills and secondly development of fundamental sport-specific skills. The environment should be fun and challenging, and skills should be achieved through process- and child-oriented playing, exercises, and small games. As in standard PE lessons, the age-related training concept allows for adjustments due to the development of the single child. The final committee proposal was accepted by the city council that also provided funding for 4 extra PE lessons per week. Children attending sports schools had a minimum of 4.5 hours PE per week, divided over at least 3 sessions and each session lasting at least 60 minutes. Twice a year, all PE teachers (i.e. normal employee PE teachers) at the sports schools took part in a service training camp with special focus on the age-related training concept. At the normal schools, the children were offered the standard 2 PE lessons per week (90 minutes in total).

### Measurements

#### Accelerometer assessed habitual physical activity

PA was assessed with the GT3X ActiGraph accelerometer (Pensacola, Florida, USA) using the vertical axis and standard filtering. After being digitalized, the accelerometer signal is passed through a filter with band limits of 0.25-2.5 HZ in order to help eliminate extraneous accelerations that were not due to human movement (e.g. vibration during passive transportation). The research staff personally delivered the accelerometers to the children at the schools, thus ensuring that children knew how to attach the device properly, placing it at the right hip using customized elastic belts. Furthermore, written information and instructions were given to children along with their parents. The children were instructed to wear the device from the time they woke up in the morning until bedtime in order to capture their entire PA for each day. The only exception was to remove the monitor when showering or swimming in order to prevent damage to the device. The children were asked to wear the accelerometers for 7 full consecutive days, thus potentially including all weekdays and a full weekend. Some children wore the instrument for more than a week since they were absent from schools when the research staff returned to pick up the accelerometers the following week. The accelerometer was set to record PA data every 2 seconds.

PA was assessed twice, once in winter 2009/10 (i.e. primo November – primo January) where children attended 1^st^ – 5^th^ grade, and once in summer/fall 2010 (i.e. medio August – primo October) where the same children now attended 2^nd^ - 6^th^ grade. Children attending matched sports schools and normal schools were measured at the exact same days.

#### Data reduction

A customized software program (Propero) was used to process accelerometer data across the domains of total time, school time, leisure time (i.e. all time out of school during weekdays), weekdays, weekends, PE lessons, and recess at a daily basis based on detailed information provided by school-class-specific time tables.

Based on manual visual inspection of all data files, a fixed time slot was applied across grades and day types to define a standard day (Additional file 2). Thereby, registrations during sleep at nights where not all children remembered to take off the instrument were avoided. Furthermore, in order to distinguish between “true” intervals of inactivity and “false” intervals of inactivity recorded when the monitor had been taken off, consecutive strings of zeros of 30 minutes or longer were interpreted as “accelerometer non-worn”. PA data was included in the analyses if the child had accumulated a minimum of 10 hours of PA per day for at least four days. Counts/min (CPM) was used as an estimate of overall mean intensity of PA, and cut-points for sedentary, light, moderate, and vigorous PA intensity levels were defined according to Evenson et al. [16].

#### Organized leisure time sports participation and bicycling

We used data collected from the SMS-track system (i.e. an automated text messaging system controlled by a web based IT-system and sent to the parents’ mobile phone) [13] to investigate if leisure time organized sports participation differed across school types. All parents were asked on a weekly basis to report the amount of organized sports participation. If the parents reported that the child had participated in organized sports, a question was sent, asking for the type of sport. The parents had 10 answering options: (1) soccer, (2) handball, (3) basketball, (4) volleyball, (5) rhythmic gymnastics, (6) tumbling, (7) swimming, (8) horse-riding, (9) dancing, and (10) other sports. Parents were asked to type the relevant number between “0” and “8”, where “0” to “7” represented the weekly number of times actually engaged in a specific sports discipline and “8” represented more than 7 times per week.

It is a well-known phenomenon that accelerometers are unable to pick up bicycling activities adequately [17]. During the week where accelerometer assessments were performed in the summer/fall in 2010, parents reported by the SMS-track system how often their child bicycled to and from school, and furthermore provided information on the time used on each trip. Answer options in minutes were: “0-5”, “5-10”, “10-15”, “15-20”, “20-25”, “25-30” and” >30”. Children’s involvement in bicycling commuting to and from school was used as a proxy for bicycling in general. The chance of biased accelerometer assessments across school types, due to bicycling, was judged based on the examination of potential differences in bicycling across school types.

#### Fitness

Cardio-respiratory fitness was determined indirectly using the Andersen Test, which is an intermittent maximal running test designed for field use and validated against directly measured maximal oxygen uptake in children [18]. Verbal encouragement was given in order to secure sufficient focus and maximal performance. Educated test-personnel registered the number of accomplished meters in order to optimize the validity of the test.

#### Anthropometrics

Body height was measured to the nearest 0.5 cm using a portable stadiometer (SECA 214, Seca Corporation, Hanover, MD), and body mass was measured to the nearest 0.1 kg using an electronic weight (Tanita Corporation, Tokyo, Japan). Assessments were performed barefooted and with the children wearing only shorts and t-shirt/undershirt. Body mass index (BMI) was calculated as weight/height^2^ (kg/m^2^) and used as a measure for body composition.

Waist circumference was measured with a Seca 201 Girth Measuring Tape to the nearest 0.5 cm at the level of the umbilicus with the child’s abdomen relaxed at the end of a gentle expiration and used as a measure for abdominal fat deposit. Two measurements were performed except in the event of a discrepancy greater than 1 cm in which case a third measurement was performed. The average of the two nearest measurements was used in the analyses.

### Statistics and data analysis

Various a priori selected accelerometer outcomes examined included: overall mean intensity of PA (i.e. CPM) and percentage of total time spend in sedentary, light, moderate, and vigorous PA intensity levels during total time all days, weekdays, weekends, school time, leisure time, PE lessons, and recess. Crude hourly overall mean PA intensity was processed to illustrate general PA levels throughout the day. Multi-level random effects models with random intercepts and maximum likelihood estimations in “XTMIXED” program (Stata version 13.0) were used to test for differences in PA levels between children attending sports schools and normal schools. A four-level model was fitted for each outcome with repeated measures (level 1) nested within children (level 2) nested within classes (level 3) nested within schools (level 4). Schools, classes, and individuals were treated as random effects, and measurement year, weekday, grade, and school type (normal schools=0 & sports schools=1) were applied as indicator variables and treated as fixed effects. All covariates were selected a priori, and since the gender x school type interaction term was a significant predictor of mean intensity of PA during school time (p=0.002), leisure time (p=0.04), and recess (p<0.001) all analyses were performed across gender. Post hoc, we added a measurement year x school type interaction term to all statistical models in order to examine if the PA level changed differently across school types over time and/or seasons.

Furthermore, based on baseline values obtained in 2008 we generated a high-BMI for age and sex group variable according to the cut-points for overweight proposed by the International Obesity Task Force [19]. Furthermore, a low cardio-respiratory fitness variable was defined as the lowest quartile of fitness for age and sex at baseline. Subsequently, we added a high-BMI x school type and a low cardio-respiratory fitness x school type interaction term in all statistical models in order to assess whether the school type specific PA level seemed to differ across subgroups of children with initial high/low BMI (high=0 & low=1) and high/low cardio-respiratory fitness (high=0 & low=1), respectively.

Based on parents’ report on their child’s discipline specific organized leisure time sports participation, we calculated children’s total number of weekly leisure time sports participation. We further estimated a “bicycling index”, as recently suggested [20] based on the parents’ report on commuter cycling and used mixed ordinal logistic regression analysis to describe possible bicycling differences between sports- and normal schools. Thus, the index was calculated by multiplying the mean minutes of the relevant bicycling category (e.g. 2.5 minutes used if parents reported a journey of 0-5 minutes and 32.5 if more than thirty minutes were reported). Only those cyclists who reported their number of cycling trips both to and from school were included in this analysis. Standard residual plots were applied to make sure that statistical model assumptions were fulfilled.

We calculated the intra-cluster correlation coefficient (ICC) as ICC= s_c_^2^/s_c_^2^+s_w_^2^) where s_c_^2^ equals variance between clusters and s_w_^2^ equals variance within clusters.

### Ethics

Participation was voluntary. All children and parents received information about the study through school meetings and written information and signed informed consent forms. The study was approved by the local scientific ethics committee (Project number: S-20080047) and performed in accordance with the Helsinki Declaration.

## Results

### Participants and wear time

At the first PA measurement year in the winter 2009/10, the mean (SD) age of participants was 9.9 (1.4) years. For further descriptive data, please see Additional file 3. Of the 1213 children who were enrolled in the CHAMPS-study DK at the first PA measurement year, 1083 children (89%) attained minimum four days of valid accelerometer recording (sample average: 6.2 (0.9) valid days). At the second PA measurement year, where 1198 children were enrolled in the study, 1047 children (87%) obtained minimum 4 valid days of monitoring (sample average: 6.1 (0.9) valid days). Of all children eligible at the two measurement years, only 34 subjects had missing PA data due to children being absent from school, download errors, or loss of instruments.

The mean (SD) daily valid wear time was 13.3 (1.3) hours with no differences present across school types (p=0.59) and no different development across school types present over time and/or seasons (p=0.74 for school type x measurement year interaction term). There were no significant differences in body weight, BMI, and cardio-respiratory fitness between children attaining and not attaining valid PA data (first assessment year: all p-values>0.53, second assessment year: all p-values>0.18). Furthermore, there were no differences in the proportion of children attaining valid PA data across school types (first assessment year: p=0.23, second assessment year: p=0.67).

### General physical activity description

**Figure 1:**
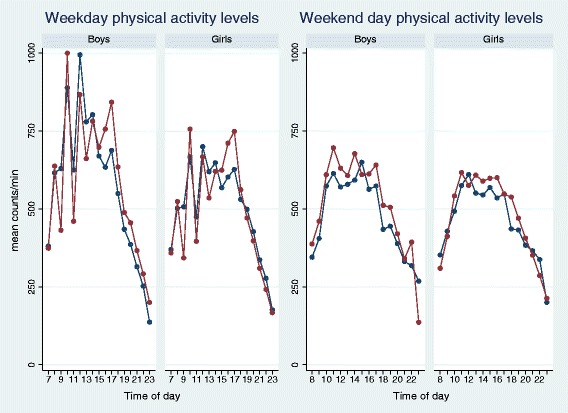
**Crude hourly overall mean total physical activity (CPM) by type of day and gender.** Blue color = sports schools, red color = normal schools. CPM: counts/min.

Based on simple descriptive data, increased PA levels were observed in boys when compared to girls (p<0.001) and in younger children when compared to older children (p<0.001) (Table 1). Additionally, crude hourly mean PA level revealed that the largest PA decline generally takes place during the afternoon, and lower PA levels were observed in weekends compared to weekdays (p<0.001) (Figure 1). The domain characterized by the highest PA level was PE lessons (boys: approx. 1510 CPM; girls: approx. 1260 CPM) followed by recess (boys: approx. 1350 CPM; girls: approx. 950 CPM), school time (boys: approx. 710 CPM; girls: approx. 550 CPM) and leisure time (boys: approx. 600 CPM; girls: approx. 540 CPM), (p<0.001 for all differences across domains except p=0.29 for differences between school time and leisure time in girls). Boys and girls who were overweight/obese or low fit at baseline displayed lower levels of overall CPM and accumulated less time in the vigorous PA level than did normal weight and normal fit children. The same PA differences were observed across these subgroups of children during PE (p<0.02 for all subgroup comparisons, except p=0.30 for differences in overall CPM between overweight/obese and non-overweight/obese girls). Furthermore, slightly increased PA levels were observed in the second measurement year compared to the first assessment year (p<0.001).

**Table 1:** **Crude physical activity levels stratified by gender and grade**

	**1** ^**st**^ **grade**	**2** ^**nd**^ **grade**	**3** ^**rd**^ **grade**	**4** ^**th**^ **grade**	**5** ^**th**^ **grade**
**Total**	*n=207*	*n=234*	*n=264*	*n=244*	*n=239*
Mean counts/min	651 (122)	598 (131)	563 (121)	550 (138)	524 (151)
Sedentary (min)	447 (39)	473 (44)	497 (44)	518 (46)	536 (53)
Light (min	256 (28)	249 (30)	237 (31)	225 (30)	219 (33)
Moderate (min)	47 (10)	44 (10)	42 (11)	40 (11)	38 (11)
Vigorous (min)	25 (9)	23 (10)	23 (9)	24 (11)	23 (11)
**Boys**	*n=94*	*n=108*	*n=140*	*n=108*	*n=110*
Mean counts/min	689 (115)	628 (140)	598 (119)	610 (144)	590 (165)
Sedentary (min)	443 (41)	469 (45)	497 (46)	505 (51)	518 (56)
Light (min	255 (27)	245 (31)	235 (32)	228 (32)	224 (34)
Moderate (min)	52 (10)	48 (11)	46 (10)	47 (11)	44 (12)
Vigorous (min)	28 (9)	26 (11)	26 (10)	28 (11)	28 (12)
**Girls**	*n=113*	*n=126*	*n=124*	*n=136*	*n=129*
Mean counts/min	620 (119)	573 (116)	524 (112)	502 (112)	468 (112)
Sedentary (min)	451 (119)	477 (44)	498 (42)	529 (39)	552 (45)
Light (min	257 (29)	253 (29)	239 (29)	223 (28)	214 (30)
Moderate (min)	42 (9)	40 (9)	36 (9)	36 (8)	33 (8)
Vigorous (min)	22 (8)	21 (8)	19 (8)	20 (8)	19 (9)

Data are mean +/- SD. Minutes in PA intensity intervals are minutes/day. Grade refers to children’s grade when measured the first time in the winter in 2009/10.

ICC for the effects of schools and classes on PA levels during total time, school time, leisure time and weekends ranged from 0.01 to 0.08 for both boys and girls. Total time and weekend time exhibited the lowest ICCs which were generally 0.01. However, during PE and recess the ICC at the school level and the class level was higher ranging from 0.04 to 0.17. School level ICCs were higher than class level ICCs during PE, while it was the other way around during recess. ICCs were generally higher in boys than in girls.

### PA differences across school types

Crude hourly mean PA levels observed in sports schools- and normal schools children, respectively, can be seen in Figure 1. Total valid wear time and crude minutes obtained in different PA intensities are presented across school types and assessment years in Additional file 4.

Boys attending sports schools were more active during school time than boys attending normal schools (β=113, p<0.001). This difference only achieved borderline level of significance in girls (β=51, p=0.065). However, in the leisure time during weekdays children who attended sports schools were less active than children who attended normal schools (girls: β=-41, p=0.004, boys: β=-72, p<0.001). Compared to children attending normal schools, less percentage of total time was spend sedentary and more percentage of time was accumulated in moderate-to-vigorous physical activity (MVPA) during school time in children who attended sports schools. However, children who attended sports schools spend more percentage of their time sedentary and accumulated less percentage of time in MVPA in their leisure time (Tables 2 and 3).

**Table 2:** **Differences in physical activity variables across different domains in boys attending sports schools and normal schools, respectively**

	**PA domain**											
	**Total time**		**School time**		**Leisure time**		**Weekend**		**PE**		**Recess**	
**PA intensity**	**β**	**P**	**β**	**P**	**β**	**P**	**β**	**P**	**β**	**P**	**β**	**P**
Overall mean (counts/min)	−10 (13)	0.452	113 (24)	<0.001*	−72 (19)	<0.001*	−24 (26)	0.369	−7 (147)	0.963	96 (80)	0.232
Sedentary time (% of total time)	0.60 (0.57)	0.288	−2.53 (1.06)	0.017*	2.3 (0.72)	0.002*	1.04 (0.97)	0.280	0.87 (4.2)	0.835	−2.42 (1.95)	0.213
Light PA (% of total time)	−0.38 (0.52)	0.458	0.54 (0.69)	0.433	−0.79 (0.64)	0.220	−0.76 (0.63)	0.230	−0.26 (1.66)	0.877	0.78 (0.92)	0.397
Moderate PA (% of total time)	−0.22 (0.20)	0.261	0.77 (0.31)	0.012*	−0.84 (0.23)	<0.001*	−0.19 (0.33)	0.562	−1.04 (1.45)	0.471	0.60 (1.10)	0.586
Vigorous PA (% of total time)	0.06 (0.11)	0.598	1.19 (0.22)	<0.001*	−0.53 (0.16)	0.001*	−0.10 (0.22)	0.638	0.20 (1.50)	0.892	1.00 (0.86)	0.243

“β” is adjusted beta-coefficients from mixed models and indicates differences in counts/min and % of total time between sports schools children and normal schools. “*” indicates significant difference between boys attending sports schools and normal schools, respectively.

**Table 3:** **Differences in physical activity variables across different domains in girls attending sports school and normal schools, respectively**

	**PA domains**											
	**Total time**		**School time**		**Leisure time**		**Weekend**		**PE**		**Recess**	
**PA intensity**	**β**	**P**	**β**	**P**	**β**	**P**	**β**	**P**	**β**	**P**	**β**	**P**
Overall mean PA (counts/min)	−10 (12)	0.410	51 (28)	0.065**	−41 (14)	0.004*	−32 (17)	0.058**	−15 (97)	0.876	−106 (88)	0.228
Sedentary time (% of total time)	0.40 (0.67)	0.553	−2.21 (1.06)	0.037*	1.81 (0.76)	0.017*	1.13 (0.60)	0.060**	0.85 (3.14)	0.787	0.95 (2.14)	0.658
Light PA (% of total time)	−0.14 (0.55)	0.803	1.28 (0.75)	0.088**	−0.88 (0.62)	0.156	−0.69 (0.55)	0.208	−0.64 (1.46)	0.664	0.55 (1.09)	0.616
Moderate PA (% of total time)	−0.19 (0.15)	0.191	0.47 (0.25)	0.063**	−0.62 (0.14)	<0.001*	−0.20 (0.18)	0.246	−0.09 (1.04)	0.931	−0.71 (0.82)	0.381
Vigorous PA (% of total time)	−0.05 (0.09)	0.572	0.46 (0.21)	0.030*	−0.32 (0.11)	0.004*	−0.24 (0.13)	0.081**	−0.34 (0.90)	0.703	−0.83 (0.74)	0.258

“β” is adjusted beta-coefficients from mixed models and indicates differences in counts/min and % of total time between sports schools children and normal schools. “*” indicates significant difference between girls attending sports schools and normal schools, respectively.

“**” indicates borderline significant difference between girls attending sports schools and normal schools, respectively.

There were no significant differences in PA levels during total time, PE, or recess between children attending sports schools and normal schools, respectively - neither in boys nor in girls. In the weekend, however, there was a tendency that the girls who attended sports schools were less active compared to the girls attending normal schools (β=-32, p=0.058). More percentage of time spend sedentary and less percentage of time accumulated in vigorous PA seemed to explain the observed differences in girls during the weekend (Tables 2 and 3).

### Organized leisure time sports participation and bicycling

Girls, but not boys, attending sports schools were less involved in leisure time organized sports participation than children who attended normal schools (boys: β=-0.2, p=0.236; girls: β=-0.4, p=0.016, where “β” equals mean weekly differences in number of organized sports participation between sports schools children and normal schools children). The proportion of cycling boys was 67% vs. 63% of girls cycling. Children who bicycled to and from schools at the sports school possessed a higher bicycling index than children who bicycled to and from schools at the normal schools (odds ratio=1.5; 95% CI: 1.1 – 2.0, odds ratio is interpreted as cycling children from sports schools having a higher bicycling index than cycling children from normal schools). The median cycling index of cyclist was 75 minutes at sports schools and 60 minutes at normal schools. No significant cycling index differences were observed between boys and girls (odds ratio=1.3; 95% CI: 0.9-1.7). The proportion of children reporting any cycling to and from school did not differ significantly across the two school types (odds ratio=0.7; 95% CI: 0.3 – 1.4) or across gender (odds ratio=0.9; 95% CI: 0.7 – 1.3).

### Physical activity differences across school types in subgroups of children classified according to weight status and cardio-respiratory fitness level at baseline

No interaction between school type and high-BMI for age and sex group was observed overall or in any domain (p>0.26 for all interaction terms), indicating that the effect of extra PE lessons did not differ across groups of children classified at baseline as normal weight and overweight/obese, respectively. Likewise, no low-fit x school type interaction was evident in boys overall or in any domain (p>0.22 for all interaction terms). However, a significant interaction was observed during PE in girls (CPM: p=0.01; vigorous PA: p=0.03) meaning that low-fit girls at sports schools recorded a higher mean PA level (i.e. 102 CPM) and more time in vigorous PA (i.e. 0.7% of PE time) compared to low-fit girls at normal schools. High-fit girls at the sports schools, on the other hand, recorded a lower mean PA level (i.e. -110 CPM) and less time in Vigorous PA (i.e. -1.15% of PE time), when compared to high-fit girls at normal schools.

### Physical activity development over time across schools types

The year x school type interaction term was significant for overall CPM (p=0.002) and vigorous PA (p=0.002) in boys, indicating that PA differences across school types were changing over time. At the first assessment year, boys who attended sports schools were more active than boys who attended normal schools, whereas it was the other way around at the second assessment year (Additional file 5). Differences across school types were however non-significant at both time points. The interaction term was also significant for CPM (p=0.002), and vigorous PA (p<0.001) and borderline significant for moderate PA (p=0.07) in boys during leisure time, reflecting that sports schools boys’ reduced PA level compared to normal schools boys’ was more evident at the second assessment year than at the first assessment year (Additional file 5). Finally, the interaction term was significant in boys for all PA outcomes during PE (p<0.03 for all interaction terms). The stratified analyses according to measurement year revealed minor and non-uniform and insignificant differences across school types at the first and second measurement occasion, respectively (all p-values>0.44). For girls, the year x school type interaction term was significant for CPM, moderate- and vigorous PA during school time (p<0.001, p=0.02, and p=0.004, respectively) and recess (p=0.004, p=0.04, and p=0.03, respectively). During PE, significant interaction terms were observed for sedentary time, light-, and moderate- PA in girls (p=0.02 for all PA intensities). The significant interaction terms which were revealed during school time were explained by decreasing PA differences observed across school types over time. Girls were significantly more active at the sports schools than girls at the normal schools during school time at the first assessment year (p=0.003) but not at the second assessment year (p=0.517). During recess at the first assessment year, sports school girls’ PA level was lower than normal schools girls’ PA level, but further reduced at the second assessment year. During PE, the differences in PA levels across school types in girls were minor and did not differ significantly at any of the two measurement occasions, although development over time were in favor for the normal schools (Additional file 5).

## Discussion

The main findings in this study were that there were no overall differences in the PA levels between children attending sports schools with mandatory trebling of PE lessons and normal schools, respectively. However, children from sports schools were more physically active during school time but less active during leisure time when compared to their peers at the normal schools, meaning that children who attended sports schools spend less time sedentary and accumulated more time in MVPA during school time but spend more sedentary and accumulated less time in MVPA during leisure time. Analyses of SMS-track data reported by the parents revealed that differences in organized sports participation contributed to the observed diverse PA levels across school types during leisure time, especially in girls. The only moderating effect of baseline status on PA differences across school types was observed during PE in girls across subgroups of cardio-respiratory fitness.

Generally, our findings support the conclusion of a small to negligible overall effect of intervention on PA of children recently reported in a systematic review and meta-analysis of controlled accelerometer-based trials [21]. Our findings also support the lately conclusion by Dobbins et al. who reported that there is evidence to suggest that school based PA interventions lead to more engagement in MVPA during school hours [10]. Based on the assumption of a standard school day of 6 hours we post hoc tried to estimate what the most noticeable differences observed across school types regarding children’s percent of total time spend in various PA intensities in the present study would transfer into if reported in absolute minutes per day. Seen in this way, sports schools boys would be estimated to accumulate 9 minutes less of sedentary behavior, but 3 and 4 minutes more of moderate and vigorous PA, respectively, when compared to normal schools boys. Similarly, sports schools girls accumulated 8 minutes less sedentary time, 4.5 minutes more time of light PA, and 2 minutes more time of both moderate and vigorous PA. Seen in the light of most PA recommendation or the daily minutes of MVPA apparently needed to prevent clustering of cardiovascular disease risk factors [22], these differences must be considered as rather limited. However, these estimated differences should be seen in the light of extrapolation of data to a full standard day and in the light of controversies linked to accelerometer assessed cut-off points for various PA intensities [23].

Finally, our findings support other results observed in the CHAMPS-study-DK. Klakk et al. previously reported that the four additional PE lessons per week did not significantly improve overall mean BMI or mean total body fat percentage in children when compared to children with two PE lessons per week [24]. Furthermore, Heidemann et al. observed that time spend in MVPA was positively associated to changes in bone mineral content, density, and area. Interesting, however, no effect on bone health of children attending a sports school could be observed [25].

### Sources of variations and domains

The weekday activity patterns observed in the present study revealed the largest decline in PA level to take place during the afternoon. Our finding of a lower PA level during weekends compared to weekdays, decreasing PA level with increasing age, and increased PA in boys compared to girls has been confirmed by others [26,27]. Additionally, we found the difference between boys and girls to be larger during school time than during leisure time. We observed PE lessons to be the domain characterized by the highest PA level, then followed by recess, school time, and leisure time, where especially boys displayed reduced PA levels during leisure time when compared to school time. This supports previous findings by Nilsson et al. who reported the mean PA level of Danish 9-year-olds to differ significantly between school time and leisure time [28]. These results thus indicate a potential for promoting PA also during leisure time, especially in boys, and furthermore point to that detailed assessments of PA levels, both between days and within days, are of importance in order to increase the understanding of the variation in PA patterns and thereby facilitate interventions targeted at increasing PA in children. It is worth noticing, however, that we observed PA levels during recess which did not differ substantially from PA levels during PE lessons. Therefore, PA interventions targeting recess specifically seems less relevant, at least in Danish children, unless more time is provided by the school for recess as the PA level is already quite high during recess and thus from a theoretical point of view difficult to lift any further.

Our findings of a lower level of PA during leisure time at sports schools compared to normal schools were, if not unexpected, not wished for. Our findings are similar to cross-sectional findings reported by Mallam et al. [29] who also observed increased school-time PA but decreased leisure-time PA in conjunction with additional PE. Our findings also confirm the results provided in a systematic review on school based PA interventions by Kriemler et al. who concluded that children’s PA level during school time can be increased but typically does not transfer into an overall positive PA effect [14]. Based on the assumption of a standard leisure time awake day of 8 hours, we post hoc tried to estimate what the observed findings regarding differences in children’s percent of total time spend in various PA intensities across school types in the present study would transfer into in terms of absolute minutes per day. Sports schools boys were estimated to accumulate 11 minutes more sedentary time and 4 and 2.5 minutes less of moderate and vigorous PA, respectively, when compared to normal schools boys. Also, sports schools girls would accumulate 9 minutes more sedentary time and 3 and 1.5 minutes less time of moderate and vigorous PA, respectively. We observed no differences in mean PA levels, or specific PA intensities levels, during PE between sports schools and normal schools. We speculate that PE offers an arena where the PA intensity is high in most children and that any further increase in average intensity could be difficult to achieve. Despite the lack of higher intensity other undisclosed benefits of being a certified sports school might have occurred such as improved quality of PA offered to the child, improved motor skills and social behavior. However, these interpretations and thoughts are solely based on speculations and beliefs and not on empirical information. It must be emphasized that the CHAMPS-study DK was conducted in a rather small community, and consequently contamination could have occurred although children with extra PE lessons and normal PE, respectively, attended separate schools. The children at normal schools who served as controls (and their teachers) might have been very motivated and eager to show that they were (also) active and that their PE lessons were fully as competent and intensive as at the sports schools. This, of course, would jeopardize the validity of the observed results.

Finally, the ICCs observed during PE and recess confirms the notion that specific schools and classes seems to play a vital role in the child’s PA behavior.

### Organized leisure time sports participation

We add to previous findings by reporting a plausible cause for the diverse PA differences observed between sports schools and normal schools children during school time and leisure time, respectively. Whether the nature of this “compensating” behavior lies with the child, its parents, or is just a spurious result (e.g. due to differences in neighborhoods as the schools were not randomized) is of course of great interest. Compensation by the individual child due to an underlying biologically controlled PA level could potentially explain our observations, and has previously been described as the “ActivityStat hypothesis” by others [30,31]. It should be emphasized, however, that evidence of biological PA determinants is not per se evidence of the existence of the ActivityStat and a form of compensation could take place even without any homeostatic regulation occurring [32]. We believe that our findings in the present study is more likely a result of parents perceiving their child as being “sufficiently active” and therefore might have grasped the opportunity for a simpler logistic solution for the entire family by not encouraging their child to participate in organized sports and/or facilitate transportation. We possess non-scientific empirical data in the form of several statements from parents to support such a theory. Therefore, we believe that compensation primarily was a result of more external oriented factors and not a biological controlled mechanism as such. Furthermore, since the extra PE lessons were added to the school curriculum as extra classes, it is likely that the children have reduced their time committed to others activities out of school. Olds et al. have previously conceptualized the trade-offs between different domains of time use as the cross-elasticity of demands, meaning that the time spend on one activity varies as the amount of time spend on another varies [33]. However, we have no obvious explanation for why this behavior should be more prevalent in girls than in boys. Regardless of the origin of the observed PA differences across specific domains, as the overall PA difference across school types (as measured by accelerometers) was almost zero a discussion of where, how, and when children should be active to achieve the largest benefits, and which benefits, is warranted. For instance, low grade in school sports has been found to associate with physical inactivity in adulthood [34]. But, if a child can improve skills by being active in the school setting, better grades and facilitating prevention of sedentary behavior also after the child has left school could maybe be the desirable result? On the other hand, organized sports participation early in life has been shown to be associated with increased PA levels in later life [35]. Consequently, the issue whether or not PA related to organized sport outside of school is maintained or relatively easy can be increased when the child leaves school is pivotal and should be carefully considered when planning future school-based PA intervention programs.

### Longitudinal observations

No clear time trend differences were observed in PA levels during PE across school types. Although no substantially altered PA patterns were revealed over time across school types, significant year x school type interaction terms were observed in other domains, generally in favor of the normal schools. As least some of the time trends which revealed decreasing PA differences during school time across school types seemed to be explained by the development of PA during recess and not during PE. We speculate that the extra PE program might have had a knock-on effect on children’s PA level in other domains, but that this knock-on effect diminished over time. This might have been explained by loss of novelty and fading enthusiasm and motivation towards the project in both teachers and children. Mean PA intensity increased from the first assessment year to the second assessment year at both sports schools and normal schools. Seasonal effects are believed, at least partly, to explain the increased PA level observed over time [36]. The observed significant year x school type interactions could also reflect seasonal variation, meaning that it is plausible that a program implying extra PE would be expressed most clearly to be effective during the season in which children are normally less active. Finally, the observed limited modifying effect over time should be cautiously interpreted, as these models were fitted post hoc as exploratory analyses. Therefore, they are suspect to random findings introduced by multiple testing, although results were generally uniform favoring normal school children’s PA level over time.

### Subgroup physical activity differences across school types

Indeed the most important question is not how to affect children who are highly active but how to affect those who are the least active and at high risk, often within an overall active and healthy population. Our BMI subgroup analyses indicated that the sports schools were not “more successful” than the normal schools in integrating the high-BMI groups of children in the PA performed. It should be emphasized, though, that the overweight/obese children at the sports schools engaged in three times as much PE (i.e. the domain where children displayed the highest PA intensity level) than the overweight children attending normal schools. Consequently, to some extent it should be considered as a positive finding that the sports schools actually succeeded in maintaining the PA intensity level in the group of overweight/obese children despite the substantially increased volume of PE. This might add to the explanation why sports schools children classified as overweight/obese at baseline in the CHAMPS-study DK benefitted in particular in terms of improved BMI when compared to baseline overweight/obese children at normal schools and also why sports schools children have been found to have a significant reduced risk of becoming overweight/obese compared to children at normal schools [24]. Similar PA levels observed across BMI subgroups at sports schools and normal schools, respectively, in the present study should be seen in the light of that participants in the CHAMPS-study DK were quite healthy, including low prevalence of overweight and obesity [24]. Behavioral modifications through fundamental movement skills program delivered in addition to the usual PE and sports classes have previously been proved to increase PA levels in Australian children characterized by an extremely high prevalence of overweight/obesity (≈47% for boys and ≈38% for girls) [37].

Although not a primary outcome in this study, and although we cannot exclude the chance that it could be a random finding as the result of multiple testing, it is of interest that low- fit girls at sports schools were more active than low-fit girls at normal schools (at sports schools low-fit girls were not significantly less active during PE than high-fit girls). This suggests that the age-related training concept and/or the education of the PE teachers were successful in increasing PA in low-fit girls, at least in the PE setting. This theory is supported by previous findings in the CHAMPS study-DK where improved cardiovascular health effects of the extra PE lessons have been observed in particular in children with a composite cardiovascular disease risk factor score above the median at baseline [38]. These findings support what has been stated by others, namely that the effect of school based preventive interventions on health outcomes is likely to be most pronounced in subgroups of individuals with low initial fitness [39].

### Bicycling

The proportion of children reporting any cycling to and from school did not differ significantly across the two school types. However, children from sports schools who used their bicycle for transportation possessed a higher bicycling index (i.e. more time used on commuter cycling) than children who bicycled at the normal schools. It should be noted though that only 578 participants had complete information on both cycling to and from school in addition to information on time spent cycling. Furthermore, the estimated bicycling index must be expected to include considerable measurement error. However, we find no reason to believe that the measurement error was systematic across school types, and proportions of children with missing cycling data for calculation of the cycling index did not differ between school types. Thus, a potential underestimation, although probably minor, of the PA level in the sports school children, especially during leisure time, should be considered when interpreting the accelerometer-based results observed in this study.

Bicycling related PA during PE and school time in general was not assessable, meaning that we can only speculate if use of bicycles differed across school types in these domains.

### Physical activity intervention programs

The PA program was limited to the PE lessons and delivered by PE teachers who attended biannually age-related training courses, and all children participated in the extra PE lessons as this was mandatory. The high participation rate (approx. 80%) in the CHAMPS-study DK indicates that families supported the idea of extra PE lessons as well as the scientifically evaluation although they were not an “active” part in the program. Included in the age-related training concept which was pivotal in the extra PE lessons was not an aim of achieving a certain amount of MVPA. A recent study by Resaland et al. demonstrated significant effects in Norwegian children’s CVD risk profiles in a school-based PA intervention of 60 min. of daily MVPA [40]. Their protocol was very focused on achieving high PA intensity levels, giving rise to the thought that a combination of the age-related training concept and intense focus on MVPA might benefit future interventions. Despite no PA level differences observed during PE, we observed that children who attended sports schools were more active and relative to total wear time accumulated less time on sedentary behaviors but more time in MVPA during school time than children who attended normal schools. Theoretically, this could be explained by the fact that children who were enrolled at sports schools were engaged in six PE lessons (4.5 hours) per week compared to only two (i.e. 1.5 hours) for children enrolled at normal schools. Intuitively, it seems that a combination of programs targeting PA through increased lengths of various high active periods during the school day (e.g. PE and recess) most likely would lift children’s PA level further, at least during school time. However, this has yet to be supported by findings in high quality studies.

### Strengths

A major strength in this study implies the high compliance of objective measurements of habitual PA (approx. 88% of all subjects provided valid PA data) at two time points in a large number of children participating in a prolonged school-based natural PA experiment. The fact that researchers had no influence on the content and intensity of PE lessons greatly strengthens the external validity of our study and increases the likelihood that similar projects can be implemented elsewhere in Denmark and perhaps even in other countries. Use of the Propero software program and access to time schedules at all schools made it possible for us to analyze PA levels specifically in a number of relevant age and school specific time slots, including leisure time, school time, recess, and PE lessons. This allowed for a more detailed description of differences in PA levels across school types. Other strengths include available information on leisure time organized sports participation, assessed consecutively on a weekly basis by SMS track system, information on commuter cycling, the inclusion of a “no-treatment” control group, and that the extra PE program was carried out by PE teachers who attended courses in the age-related training concept.

### Limitations

A limitations to this study is the lack of baseline accelerometer assessed habitual PA level and leisure time organized sports participation before the trebling of PE lessons was introduced at the sports schools, which was due to a very short notice on the initiation of the natural experiment in the municipality of Svendborg. Thus we are not able to determine causality and estimate true intervention effect sizes. Six schools volunteered to be sports schools and subsequently 4 schools, matched on socio-economic status of their uptake area and size, were selected to serve as control schools (i.e. normal schools). Sports schools and normal schools, serving as controls, were matched not randomized, which would have eliminated potential selection bias and strengthen the external validity of our results. Matching on socio-economic status of the uptake area and school size might not totally have eliminated all relevant differences between school types, including PA climate and overall beliefs and perceptions towards children’s healthy behavior. However, based on information from the National Danish Registry of Statistics there were no significant differences in household income (p=0.89) and educational level (p=0.81) between sports schools and normal schools, thus indicating that proper matching of schools in terms of socio-economic status occurred. The fact that two normal schools served as a match for two pairs of sports schools should be seen as a limitation to the present study, although the uptake area of children was shared within the two pairs of sports schools. There were no differences in BMI across school types at baseline (p=0.18). However, our findings should be seen in the light of that we observed slightly higher cardio-respiratory fitness performance at baseline in girls who attended sports schools compared to girls who attended normal schools (+17 meter performance in the Andersen Test, p=0.01) (data not shown).

The inability to capture cycling, swimming, and load-bearing activities correctly by accelerometry is a limitation in this study. However, with the exception of the previously mentioned bicycling index differences we have no reason to believe that comparability between sports schools and normal schools should be affected due to this.

Any conclusions in terms of what might have caused PA to increase or decrease at the sports schools is complicated due to the fact that the extra PE program was introduced in a natural experiment. Finally, generalizability of the results may not extend beyond the type of population sampled in the present study (small town with surrounding rural district in Denmark).

## Conclusions

Children from the sports schools were more physically active during school time but less active in their leisure time when compared to their peers from normal schools. Analyses of SMS-track data revealed that reduced PA levels during leisure time in sports schools girls was explained, at least partly, by less organized sports participation. Baseline status of cardio-respiratory fitness was observed to modify school type differences in PA levels during PE in girls. There were no significant differences in overall PA levels, PA levels during recess, PE, or during the weekend across the two school types. No clear PA trend differences were observed across school types from the first to second assessment year.

## Additional files

Additional file 1: Figure S1. Participants flow in the CHAMPS-study DK. Description of data: Charge represents overall flow of participants in the CHAMPS-study DK during a 3-year period and also illustrates when accelerometer assessments were performed. Additional file 2: Table S1. Standard waking hour definition across different age groups and weekdays. Description of data: Results are based on manual visual inspection of all data files. Additional file 3: Table S2. Age, anthropometrics and cardio-respiratory fitness assessed at children’s schools in March/April 2010 between the two accelerometer assessment periods. Data are crude means (SD) and proportions presented by gender and grade. Description of data: NW: normal weight, OW: overweight, OB: obese, CRF: cardio-respiratory fitness. Only children who were tested at their school in spring 2010 and attained valid accelerometer assessments are included in the table. Due to some children possessing missing values in some tests and some children being absent when testing was performed at the schools, “n” differs across variables presented and across presented variables and physical activity outcomes reported in the study. Additional file 4: Table S3. Boys’ total valid wear time and total minutes accumulated in various physical intensity levels across school types and assessment years. **Table S4.** Girls’ total valid wear time and total minutes accumulated in various physical intensity levels across school types and assessment years. Description of data: All data are crude means and (SD) reported in minutes. “1” denotes first assessment year, “2” denotes second assessment year. Additional file 5: Figure S2. Illustrations of significant interactions between physical activity levels across school type and assessment year. Description of data: Margins plots illustrating interactions for counts/min (CPM). PE: physical education. Results are from random effects models (“XTMIXED” in STATA) with both the main effects and the interaction terms included in the models. Physical activity assessments were performed in winter 2009/10 and in summer/fall 2010, respectively. Values are adjusted means with 95% CI. Please note that y-axes do not start at zero.
