# Supplementary figures and images for: Do extra compulsory physical education lessons mean more physically active children - findings from the childhood health, activity, and motor performance school study Denmark (The CHAMPS-study DK)
Source: Int J Behav Nutr Phys Act. 2014 Sep 24;11:121. doi: 10.1186/s12966-014-0121-0 (PMC4180151; doi:10.1186/s12966-014-0121-0)

**Supplementary figure 1.** Participants flow in the CHAMPS-study DK

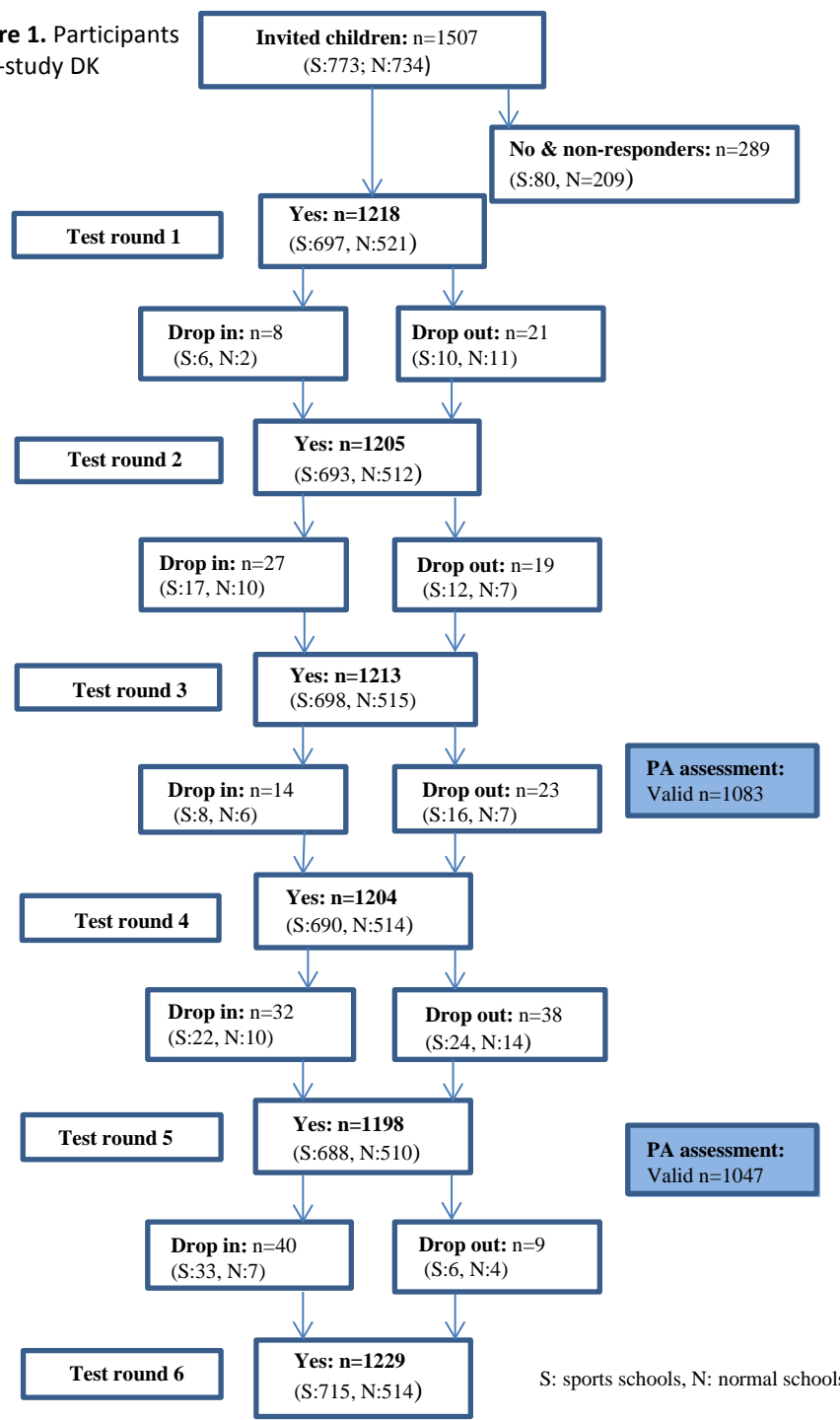

Supplement: Additional file 1: Figure S1. — Participants flow in the CHAMPS-study DK. Description of data: Charge represents overall flow of participants in the CHAMPS-study DK during a 3-year period and also illustrates when accelerometer assessments were performed. [file 12966_2014_121_MOESM1_ESM.pdf]
