# Supplementary material for: Do extra compulsory physical education lessons mean more physically active children - findings from the childhood health, activity, and motor performance school study Denmark (The CHAMPS-study DK)
Source: Int J Behav Nutr Phys Act. 2014 Sep 24;11:121. doi: 10.1186/s12966-014-0121-0 (PMC4180151; doi:10.1186/s12966-014-0121-0)
Supplement: Additional file 2: Table S1. — Standard waking hour definition across different age groups and weekdays. Description of data: Results are based on manual visual inspection of all data files. [file 12966_2014_121_MOESM2_ESM.pdf]

Supplementary table 1. Standard waking hour definition across different age groups and weekdays

Grade	Mon-Thurs	Friday	Saturday	Sunday
1st grade	06.30-20.30	06.30-22.00	08.00-22.00	08.00-20.30
2nd grade	06.30-21.00	06.30-22.00	08.00-22.00	08.00-21.00
3rd grade	06.30-21.00	06.30-22.30	08.00-22.30	08.00-21.00
4th grade	06.30-21.30	06.30-22.30	08.00-22.30	08.00-21.30
5th grade	06.30-22.00	06.30-23.00	08.00-23.00	08.00-22.00
6th grade	06.30-22.00	06.30-23.00	08.00-23.00	08.00-22.00
