# Supplementary material for: Do extra compulsory physical education lessons mean more physically active children - findings from the childhood health, activity, and motor performance school study Denmark (The CHAMPS-study DK)
Source: Int J Behav Nutr Phys Act. 2014 Sep 24;11:121. doi: 10.1186/s12966-014-0121-0 (PMC4180151; doi:10.1186/s12966-014-0121-0)
Supplement: Additional file 3: Table S2. — Age, anthropometrics and cardio-respiratory fitness assessed at children’s schools in March/April 2010 between the two accelerometer assessment periods. Data are crude means (SD) and proportions presented by gender and grade. Description of data: NW: normal weight, OW: overweight, OB: obese, CRF: cardio-respiratory fitness. Only children who were tested at their school in spring 2010 and attained valid accelerometer assessments are included in the table. Due to some children possessing missing values in some tests and some children being absent when testing was performed at the schools, “n” differs across variables presented and across presented variables and physical activity outcomes reported in the study. [file 12966_2014_121_MOESM3_ESM.pdf]

Supplementary table 2. Age, anthropometrics and cardio-respiratory fitness assessed at children's schools in March/April 2010 between the two accelerometer assessment periods. Data are crude means (SD) and proportions presented by gender and grade

	**1st grade**	**n**	**2nd grade**	**n**	**3rd grade**	**n**	**4th grade**	**n**	**5th grade**	**n**
**Total**										
Age (years)	7.9 (0.4)	196	8.9 (0.3)	222	9.9 (0.4)	250	10.9 (0.3)	236	11.9 (0.4)	225
Height (cm)	129 (6)	187	136 (6)	213	141 (7)	234	146 (6)	226	152 (7)	213
Weight (kg)	26.9 (4.0)	187	31.1 (5.5)	213	34.2 (6)	234	37.8 (6.9)	226	42.3 (8.5)	213
NW/OW/OB (n(%))	171 (91) / 14 (7) / 2 (1)	187	188 (88) / 20 (11) / 5 (3)	213	202 (86) / 30 (13) / 2 (1)	234	201 (89) / 21 (9) / 4 (2)	226	188 (88) / 23 (11) / 2 (1)	213
Fitness (m)	895 (80)	182	927 (90)	205	960 (109)	215	992 (102)	206	1010 (97)	198
**Boys**										
Age (years)	7.9 (0.4)	89	9.0 (0.3)	100	10.0 (0.4)	133	10.9 (0.3)	103	11.9 (0.4)	106
Height (cm)	130 (6)	85	137 (6)	98	142 (7)	123	147 (7)	98	153 (7)	100
Weight (kg)	27.1 (4)	85	31.3 (5.0)	98	34.6 (6.1)	123	38.0 (7.0)	98	42.2 (8.5)	100
NW/OW/OB (n(%))	80 (94) / 5 (6) / 0 (0)	85	89 (91) / 9 (9) / 0 (0)	98	108 (88) / 14 (11) / 1 (1)	123	90 (92) / 5 (5) / 3 (3)	98	90 (90) / 10 (10) / 0 (0)	100
Fitness (m)	919 (83)	82	963 (86)	93	1002 (108)	111	1040 (99)	90	1043 (106)	93
**Girls**										
Age (years)	7.8 (0.4)	107	8.9 (0.3)	122	9.9 (0.3)	117	10.8 (0.3)	133	11.8 (0.4)	119
Height (cm)	128 (6)	102	135 (6)	115	139 (6)	111	146 (6)	128	151 (7)	113
Weight (kg)	26.8 (4.2)	102	30.9 (5.9)	115	33.7 (6.5)	111	37.7 (6.9)	128	42.1 (8.5)	113
NW/OW/OB (n(%))	91 (89) / 9 (9) /2 (2)	102	99 (86) / 11 (10) / 5 (3)	115	94 (85) / 16 (14) / 1 (1)	111	111 (87) / 16 (13) / 1 (1)	128	98 (87) / 13 (12) / 2 (2)	113
CRF (m)	875 (73)	100	898 (83)	112	915 (90)	104	954 (89)	116	980 (78)	105

NW: normal weight, OW: overweight, OB: obese, CRF: cardio-respiratory fitness. Only children who were tested at their school in spring 2010 and attained valid accelerometer assessments are included in the table. Due to some children possessing missing values in some tests and some children being absent when testing was performed at the schools, "n" differs across variables presented and across presented variables and PA outcomes reported in the study
