# Supplementary material for: Do extra compulsory physical education lessons mean more physically active children - findings from the childhood health, activity, and motor performance school study Denmark (The CHAMPS-study DK)
Source: Int J Behav Nutr Phys Act. 2014 Sep 24;11:121. doi: 10.1186/s12966-014-0121-0 (PMC4180151; doi:10.1186/s12966-014-0121-0)
Supplement: Additional file 4: Table S3. — Boys’ total valid wear time and total minutes accumulated in various physical intensity levels across school types and assessment years. Table S4. Girls’ total valid wear time and total minutes accumulated in various physical intensity levels across school types and assessment years. Description of data: All data are crude means and (SD) reported in minutes. “1” denotes first assessment year, “2” denotes second assessment year. [file 12966_2014_121_MOESM4_ESM.pdf]

Supplementary table 3. Boys' total valid wear time and total minutes accumulated in various physical activity intensity levels across school types and assessment years

	**Total time**	**School time**	**Leisure time**	**Weekend**	**PE**	**Recess**
Normal schools	*1: n=226*<br>*2: n=212*	*1: n=226*<br>*2: n=212*	*1: n=226*<br>*2: n=212*	*1: n=194*<br>*2: n=182*	*1: n=217*<br>*2: n=196*	*1: n=226*<br>*2: n=212*
**Total wear time**						
1	4863 (879)	1398 (234)	2426 (411)	1163 (382)	90 (14)	263 (46)
2	4860 (877)	1420 (206)	2366 (413)	1213 (383)	89 (6)	272 (40)
**Sedentary**						
1	2969 (595)	806 (170)	1486 (276)	757 (263)	29 (12)	89 (34)
2	2941 (652)	846 (163)	1417 (326)	762 (269)	31 (16)	98 (33)
**Light**						
1	1452 (346)	444 (103)	721 (186)	323 (126)	35 (8)	110 (27)
2	1445 (323)	434 (85)	705 (173)	348 (133)	34 (9)	115 (25)
**Moderate**						
1	291 (89)	94 (29)	147 (53)	55 (31)	14 (5)	40 (16)
2	296 (86)	90 (25)	150 (53)	64 (34)	13 (6)	37 (13)
**Vigorous**						
1	151 (67)	53 (25)	72 (39)	29 (24)	12 (7)	24 (15)
2	178 (80)	50 (25)	94 (52)	39 (30)	10 (7)	22 (15)
Sports schools	*1: n=284*<br>*2: n=257*	*1: n=284*<br>*2: n=255*	*1: n=284*<br>*2: n=255*	*1: n=256*<br>*2: n=234*	*1: n=284*<br>*2: n=253*	*1: n=284*<br>*2: n=255*
**Total wear time**						
1	4982 (834)	1541 (235)	2297 (390)	1227 (361)	242 (58)	285 (48)
2	4906 (812)	1520 (239)	2244 (388)	1220 (377)	238 (51)	280 (47)
**Sedentary**						
1	3061 (616)	845 (174)	1455 (305)	813 (256)	79 (33)	89 (36)
2	3007 (624)	871 (180)	1404 (326)	776 (263)	104 (51)	95 (36)
**Light**						
1	1467 (308)	503 (102)	660 (156)	330 (123)	98 (25)	125 (29)
2	1444 (329)	473 (103)	650 (158)	346 (145)	84 (24)	120 (29)
**Moderate**						
1	287 (83)	115 (32)	121 (44)	55 (32)	36 (12)	44 (16)
2	282 (87)	107 (33)	119 (45)	62 (37)	27 (13)	40 (16)
**Vigorous**						
1	166 (73)	78 (32)	62 (37)	29 (25)	33 (15)	28 (17)
2	174 (79)	70 (31)	71 (42)	37 (29)	22 (15)	26 (17)

All data are crude means and (SD) reported in minutes. "1" denotes first assessment year, "2" denotes second assessment year

Supplementary table 4. Girls' total valid wear time and total minutes accumulated in various physical activity intensity levels across school types and assessment years

	**Total time**	**School time**	**Leisure time**	**Weekend**	**PE**	**Recess**
Normal schools	*1: n=231*<br>*2: n=234*	*1: n=231*<br>*2: n=234*	*1: n=231*<br>*2: n=234*	*1: n=206*<br>*2: n=194*	*1: n=220*<br>*2: n=219*	*1: n=231*<br>*2: n=234*
**Total wear time**						
1	4884 (861)	1389 (250)	2446 (360)	1136 (376)	90 (13)	261 (49)
2	4865 (875)	1437 (215)	2377 (392)	1223 (383)	89 (6)	275 (42)
**Sedentary**						
1	3071 (651)	882 (202)	1508 (280)	733 (265)	34 (11)	114 (41)
2	3086 (646)	944 (181)	1458 (286)	794 (277)	38 (14)	126 (37)
**Light**						
1	1453 (315)	406 (88)	748 (162)	327 (126)	35 (8)	108 (23)
2	1425 (324)	394 (86)	734 (175)	348 (132)	34 (8)	109 (24)
**Moderate**						
1	236 (72)	65 (21)	125 (41)	50 (29)	11 (4)	26 (10)
2	226 (76)	64 (22)	118 (47)	51 (30)	10 (5)	29 (14)
**Vigorous**						
1	124 (62)	35 (16)	64 (36)	26 (25)	9 (6)	13 (9)
2	128 (64)	36 (21)	67 (38)	29 (25)	8 (5)	15 (13)
Sports schools	*1: n=342*<br>*2: n=344*	*1: n=341*<br>*2: n=344*	*1: n=341*<br>*2: n=344*	*1: n=305*<br>*2: n=306*	*1: n=341*<br>*2: n=342*	*1: n=341*<br>*2: n=344*
**Total wear time**						
1	4933 (799)	1547 (221)	2293 (352)	1186 (371)	243 (54)	286 (47)
2	4939 (859)	1539 (234)	2289 (379)	1204 (389)	242 (54)	285 (50)
**Sedentary**						
1	3107 (627)	931 (184)	1451 (288)	784 (269)	92 (32)	122 (43)
2	3122 (642)	965 (176)	1440 (291)	774 (276)	112 (49)	130 (38)
**Light**						
1	1466 (301)	478 (96)	685 (146)	330 (122)	96 (27)	121 (29)
2	1467 (331)	453 (103)	692 (168)	350 (134)	87 (26)	117 (31)
**Moderate**						
1	235 (70)	87 (27)	105 (35)	49 (27)	30 (12)	29 (14)
2	223 (69)	77 (26)	100 (36)	51 (29)	24 (13)	25 (11)
**Vigorous**						
1	125 (56)	51 (22)	52 (30)	24 (19)	25 (13)	15 (10)
2	127 (59)	45 (23)	57 (33)	28 (24)	18 (12)	13 (11)

All data are crude means and (SD) reported in minutes. "1" denotes first assessment year, "2" denotes second assessment year
