# Supplementary material for: Do extra compulsory physical education lessons mean more physically active children - findings from the childhood health, activity, and motor performance school study Denmark (The CHAMPS-study DK)
Source: Int J Behav Nutr Phys Act. 2014 Sep 24;11:121. doi: 10.1186/s12966-014-0121-0 (PMC4180151; doi:10.1186/s12966-014-0121-0)

Supplementary figure 2. Illustrations of significant interactions between physical activity levels across school type and assessment year

Overall CPM in boys

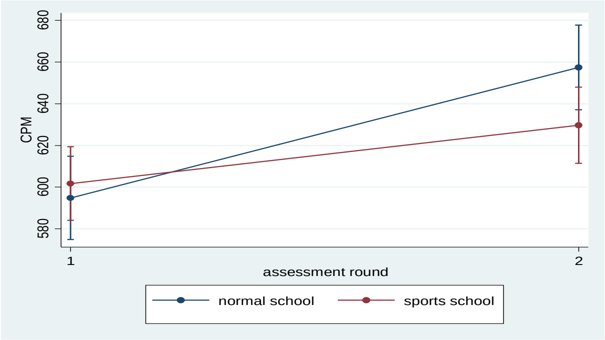

Leisure-time CPM in boys

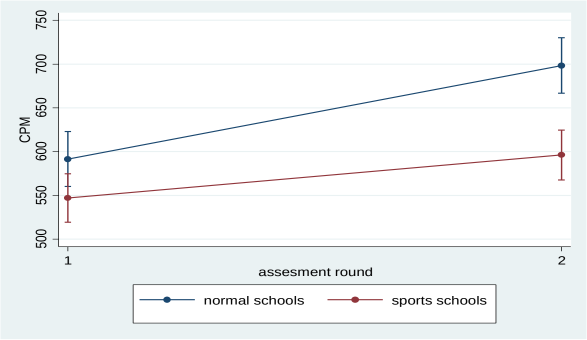

School time CPM in girls

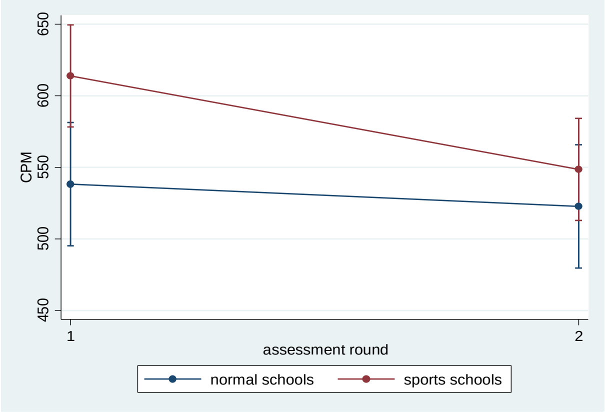

Recess CPM in girls

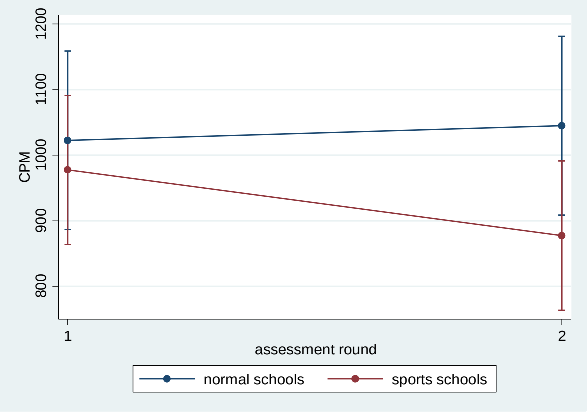

PE CPM in girls

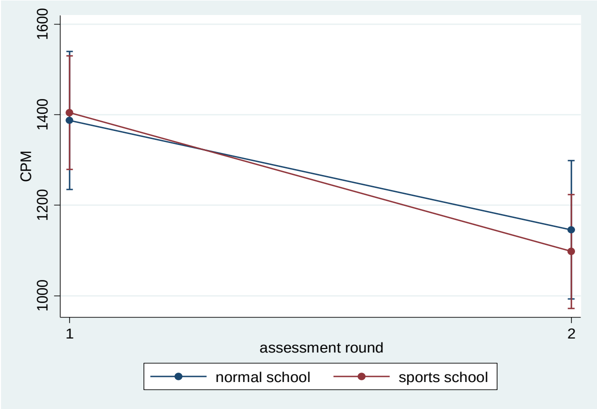

Supplement: Additional file 5: Figure S2. — Illustrations of significant interactions between physical activity levels across school type and assessment year. Description of data: Margins plots illustrating interactions for counts/min (CPM). PE: physical education. Results are from random effects models (“XTMIXED” in STATA) with both the main effects and the interaction terms included in the models. Physical activity assessments were performed in winter 2009/10 and in summer/fall 2010, respectively. Values are adjusted means with 95% CI. Please note that y-axes do not start at zero. [file 12966_2014_121_MOESM5_ESM.pdf]
